# Predictors of Financial Toxicity Trajectories in Patients With Pancreatic Cancer: A Latent Class Growth Analysis

**DOI:** 10.1002/cam4.70875

**Published:** 2025-05-04

**Authors:** Li Xiaoxuan, Zhang Shuo, Li Shanshan, Yu Mengxia, Yao Tianying, Shen Yuxin, Li Jiang, Chen Mingxia

**Affiliations:** ^1^ The First College of Clinical Medical Science China Three Gorges University Yichang China; ^2^ School of Nursing Nanjing Medical University Nanjing China

**Keywords:** financial toxicity, influencing factors, latent category growth model, pancreatic cancer

## Abstract

**Importance:**

Pancreatic cancer patients face varying medical expenses at different stages of treatment, resulting in dynamic changes in their financial toxicity. Longitudinal data collection is necessary to characterize the trajectory of these financial toxicity changes.

**Objective:**

To Explore Potential Trajectories and Influencing Factors of Financial Toxicity among Pancreatic Cancer Patients.

**Design:**

This was a prospective observational research study performed according to STROBE Checklist.

**Setting and Participants:**

From August 2022 to August 2023, we conducted inpatient data collection from pancreatic cancer patients in three hospitals in Jiangsu Province.

Main Outcomes and Measures.

The COST scale was employed to investigate financial toxicity at four time points: upon admission (T0), at discharge (T1), 3 months post‐discharge (T2), and 6 months post‐discharge (T3). A latent growth model was utilized to classify the trajectories of financial toxicity and explore its influencing factors.

**Results:**

The identification of financial toxicity trajectories among pancreatic cancer patients revealed three potential categories: a high‐risk‐financial toxicity stable group (19.69%), a moderate‐risk‐financial toxicity stable group (56.37%), and a low‐risk‐financial toxicity stable group (23.94%). Logistic regression analysis showed that, compared to the low‐risk‐financial toxicity stable group, the primary influencing factors for the high‐risk‐financial toxicity stable group were self‐efficacy and total out‐of‐pocket medical expenses. In contrast to the low‐risk‐financial toxicity stable group, the moderate‐risk‐financial toxicity stable group was influenced by self‐efficacy and total monthly household income. When comparing the moderate‐risk‐financial toxicity stable group to the high‐risk‐financial toxicity stable group, factors such as ACCI, employment status, total out‐of‐pocket medical expenses, and distance to healthcare facilities emerged as significant.

**Conclusions and Relevance:**

The financial toxicity among pancreatic cancer patients has been categorized into three distinct trajectory patterns, each exhibiting significant population heterogeneity. It is imperative that we acknowledge the profound impact of financial toxicity on pancreatic cancer patients and strengthen relevant preventive and control measures to enhance their quality of life and therapeutic outcomes.

## 1 Introduction

Pancreatic cancer (PC), a highly aggressive malignancy with complex treatment protocols and severe postoperative complications, imposes substantial financial burdens globally [1, 2, 3, 4, 5, 6, 7]. In China, PC accounts for 2.3% of all cancers, with treatment costs escalating due to surgery, chemoradiation, and novel therapies [8, 9]. According to Article 28 of the “Social Insurance Law of the People's Republic of China”, eligible medical expenses, including those for drugs, diagnostics, treatments, and medical services listed in the essential medical insurance, can be reimbursed from the basic medical insurance fund. This reimbursement covers a portion of the diagnostic, treatment, and medication costs for pancreatic cancer patients. Despite medical insurance reforms under Article 28 of China's Social Insurance Law—covering 95% of the population—many patients face persistent financial toxicity (FT) [10], defined as financial strain from direct medical expenses (e.g., out‐of‐pocket costs) and indirect losses (e.g., unemployment) [11]. Studies report a 91% FT incidence among PC patients, significantly higher than other cancers, exacerbated by productivity declines and prolonged unemployment [12, 13, 14, 15, 16].

Treatment costs peak during the initial 6 months, particularly in surgical and early treatment phases, before declining in stable periods. Hospitalization costs correlate with disease severity, comorbidities, and complications [9, 17, 18, 19]. To address gaps in understanding dynamic FT trajectories, this longitudinal study employs Latent Class Growth Modeling (LCGM) to integrate subjective (e.g., psychological distress) and objective (e.g., expenses) FT dimensions, guided by Jones'Model of Financial Burden [20, 21].

Aligned with national efforts to optimize insurance policies, this research highlights the need for tailored cost‐management strategies, especially during high‐expense phases, to mitigate catastrophic spending and improve care continuity. Findings may inform broader policies addressing financial challenges in oncology and other high‐cost medical fields.

## 2 Method

### 2.1 Participant Recruitment and Recruitment Procedure

This prospective observational research has been conducted according to the Strengthening the Reporting of Observational studies in Epidemiology (STROBE) Checklist. The study has conformed with the principles outlined in the Declaration of Helsinki and has been approved by the Ethics Committee of Nanjing Medical University, China [(2022)797]. The convenience sampling method has been adopted to recruit the patients. Data collection for the study has been conducted from August 2022 to August 2023. The data has been collected by two designated members of the research team, who have administered the financial toxicity survey to pancreatic cancer patients at four time points: at admission (T0), at discharge (T1), 3 months after discharge (T2), and 6 months after discharge (T3). The study has followed patients for financial toxicity from admission to hospital until 6 months after discharge. Additionally, information on influencing factors has been gathered. The follow‐up data collection has been conducted via telephone, following a unified methodology and standards to ensure the accuracy and reliability of the measurements.

A minimum sample of 125 was required (25 predictors × 5). Accounting for 30% attrition, 163 participants were targeted. In various studies on latent variable models, the calculation methods of sample size are different. Some studies have shown that at least 250 samples are needed when the number of categories is 5 [22]. To enhance robustness, we enrolled 340 patients, exceeding thresholds for multivariate analysis. The inclusion, exclusion, and withdrawal criteria for this study are as follows: (1)Inclusion criteria: (1) confirmed PC diagnosis via imaging/histopathology, (2)cognitive capacity to consent, (3) expected survival > 6 months (assessed by oncologists using ECOG performance status). Exclusion criteria: concurrent malignancies or severe cognitive impairment. (3)Withdrawal criteria: Participants who refuse to complete the questionnaire; invalid questionnaires due to incomplete responses, patterned answering, etc.; participants who withdraw midway; and participants who die due to their illness and are unable to complete the survey.

### 2.2 Measures

Setting: From August 2022 to August 2023, we conducted inpatient data collection from pancreatic cancer patients in three hospitals in Jiangsu Province.

#### 2.2.1 Model of Financial Burden After Cancer Diagnosis(FBCD)

This study was guided by the “Model of Financial Burden after Cancer Diagnosis” proposed by Jone et al. in 2020 [21]. This model was divided into components of financial toxicity, its causes, moderating factors, and the adverse consequence arising therefrom. Compared with other models, the content was more comprehensive [23, 24].

#### 2.2.2 Sociodemographic and Clinical Characteristics

The sociodemographic characteristics encompass gender, age, profession, employment status, monthly household income (yuan/month), quantity of children, primary caregiver, education level, marital status, housing type, distance to hospital (km), payment methods, commercial insurance, loan, and the main breadwinner of the family. The clinical characteristics comprise cancer staging, operation history, treatment on initial admission, treatment after discharge, entering ICU or not, number of complications, and length of stay (days).

#### 2.2.3 Financial Toxicity

This study intends to adopt the COST‐PROM scale, developed by scholars from the University of Chicago in 2014, to measure the severity of financial toxicity among cancer patients [25]. The COST‐PROM scale comprises 11 items, divided into three dimensions: financial expenditure (1 item), financial resources (2 items), and psychological reactions (8 items). The scale has a high reliability; Cronbach's α coefficient is 0.90. Factor analysis showed that the scale extracted a common factor that could explain 93% of the total variation, indicating that the scale had high structural validity. This study utilizes this scale to track the financial toxicity of pancreatic cancer patients at admission, discharge, 3 months post‐discharge, and 6 months post‐discharge. Financial toxicity was determined when the scale score was < 26 [26].

#### 2.2.4 Age‐Adjusted Charlson Comorbidity Index

Age‐adjusted Charlson Comorbidity Index (ACCI) is a quantitative assessment tool that comprehensively considers the impact of patient age and comorbidities on disease prognosis [27, 28, 29, 30]. Previous studies investigating factors influencing patients' financial toxicity often included the presence or absence of comorbidities as independent variables in the model analysis [31]. However, this approach may not fully capture the complex effects of comorbidities on financial toxicity. ACCI takes into account not only the number of comorbidities but also their severity in influencing financial toxicity.

#### 2.2.5 The General Perceived Self‐Efficacy Scale (GSES)

The GSES was developed by Schwarze in Germany [32]. This scale consists of 10 items, with a 4‐point rating scale ranging from “not at all true” to “exactly true,” assigned scores of 1 to 4 respectively. The total score is calculated as the sum of the ratings multiplied by the number of items (ranging from 10 to 40), with higher scores indicating a higher level of self‐efficacy. The Chinese version was Sinicized by Wang Caikang. The scale has been widely verified in Chinese population and has high reliability and validity [33].

### 2.3 Data Analysis

The sociodemographic and disease‐related data of pancreatic cancer patients were analyzed using SPSS 26.0. Continuous variables were described by mean ± SD or median, and categorical variables by frequency/percentage. Baseline characteristics were compared using *t*‐tests/ANOVA and Chi‐square/Fisher's exact tests. LCGM in Mplus 8.3 identified FT trajectories based on FT scores. Optimal model fit was achieved using Akaike Information Criterion (AIC), Bayesian Information Criterion (BIC), Sample Size‐Adjusted Bayesian Information Criterion (aBIC), Entropy, Bootstrap Likelihood Ratio Test(BLRT), Bootstrap Likelihood Ratio Test(BLRT) [22]. Significant factors were identified via univariate analysis and further examined via multivariate logistic regression (*p* < 0.05).

## 3 Results

### 3.1 Analysis of General Information and Lost‐To‐Follow‐Up Cases Among Pancreatic Cancer Patients

The enrollment of pancreatic cancer patients began in August 2022 and concluded in August 2023, with follow‐up initiated immediately after enrollment. On the day of admission (T0), a general information survey was conducted. This study collected 340 incident pancreatic cancer patients who met the inclusion and exclusion criteria and provided informed consent to participate. Three follow‐up surveys were conducted: at discharge (T1), 3 months post‐discharge (T2), and 6 months post‐discharge (T3). In total, 259 patients completed all follow‐up visits, while 81 patients were lost to follow‐up, resulting in an overall loss to follow‐up rate of 23.82%. The main reasons for loss to follow‐up were: incorrect phone number in 5 cases (6.17%), multiple rejections or refusals to participate in the survey in 14 cases (17.28%), and patient death in 62 cases (76.55%). A comparative analysis of the data from the 259 patients who completed follow‐up and the 81 patients lost to follow‐up revealed no statistically significant difference in the distribution between the two groups (*p* > 0.05), as shown in Table 1.

**TABLE 1:** Analysis of general information and lost‐to‐follow‐up cases among pancreatic cancer patients.

Variable	Groups	Completed follow‐up patients(*n* = 259)	Lost patient (*n* = 81)	*p*
		*n* (%)/ $\bar{X}$ ± SE	*n* (%)/ $\bar{X}$ ± SE	
Age	< 60	94 (36.3%)	26 (32.1%)	0.494
	60–80	156 (60.2%)	50 (61.7%)	
	> 80	9 (3.5%)	5 (6.2%)	
Gender	Male	146 (56.4%)	41 (50.6%)	0.373
	Female	113 (43.6%)	40 (49.4%)	
Profession	Farmer	59 (22.8%)	22 (27.2%)	0.506
	Work	138 (53.3%)	46 (56.8%)	
	Cadre	6 (2.3%)	1 (1.2%)	
	Others	56 (21.6%)	12 (14.8%)	
Employment status	Full‐time job	54 (20.8%)	13 (16.0%)	0.801
	Part‐time job	8 (3.1%)	3 (3.7%)	
	Retried	125 (48.3%)	43 (53.1%)	
	Unemployed	72 (27.8%)	22 (27.2%)	
Payment methods	New rural cooperative medical (NCM)	96 (37.1%)	30 (37.0%)	0.880
	Medical insurance for urban employees	130 (50.2%)	39 (48.1%)	
	The medical insurance for urban residents	31 (12.0%)	12 (14.8%)	
	Poverty relief insurance	1 (0.4%)	0 (0.0%)	
	Self‐paying	1 (0.4%)	0 (0.0%)	
Monthly household income (yuan/month)	< 5000	44 (17.0%)	12 (14.8%)	0.869
	5000–9999	117 (45.2%)	36 (44.4%)	
	10,000–14,999	74 (28.6%)	23 (28.4%)	
	≥ 15,000	24 (9.3%)	10 (12.3%)	
Quantity of children	0	3 (1.2%)	2 (2.5%)	0.566
	1	112 (43.2%)	37 (45.7%)	
	≥ 2	144 (55.6%)	42 (51.9%)	
Education level	Primary and below	88 (34.0%)	30 (37.0%)	0.085
	Junior high school	76 (29.3%)	27 (33.3%)	
	Junior high school	54 (20.8%)	11 (13.6%)	
	University	40 (15.4%)	13 (16.0%)	
	Postgraduate and above	1 (0.4%)	0 (0.0%)	
Marital status	Spinsterhood	1 (0.4%)	2 (2.5%)	0.642
	Married	227 (87.6%)	67 (82.7%)	
	Divorced	6 (2.3%)	0 (0.0%)	
	Widowed	25 (9.7%)	12 (14.8%)	
Primary caregiver	Wife/husband	112 (43.2%)	29 (35.8%)	0.486
	Sons and daughters	133 (51.4%)	46 (56.8%)	

	Nursing workers	3 (1.2%)	2 (2.5%)	
Others	11 (4.2%)	4 (4.9%)		
Housing type	Live with spouse	131 (50.6%)	42 (51.9%)	0.737
	Live with children	125 (48.3%)	39 (48.1%)	
	Others	3 (1.2%)	0 (0.0%)	
Cancer stage	1 ~ 2	146 (56.4%)	43 (53.1%)	0.604
	3 ~ 4	113 (43.6%)	38 (46.9%)	
Operation history	≥ 1	107 (41.3%)	42 (51.9%)	0.098
	0	152 (58.7%)	39 (48.1%)	
Distance to hospital (Km)	< 100	110 (42.5%)	36 (44.4%)	0.944
	100–500	127 (49.0%)	39 (48.1%)	
	> 500	22 (8.5%)	6 (7.4%)	
The main breadwinner of the family	Yes	114 (44.0%)	30 (37.0%)	0.303
	No	145 (56.0%)	51 (63.0%)	
Loan	Yes	22 (8.5%)	10 (12.3%)	0.382
	No	237 (91.5%)	71 (87.7%)	
Commercial insurance	Yes	42 (16.2%)	21 (25.9%)	0.070
	No	217 (83.8%)	60 (74.1%)	
ACCI		2.67 ± 0.08	3.52 ± 0.14	0.351

### 3.2 Characteristic Analysis of Three Categories of Financial Toxicity in Patients With Pancreatic Cancer

#### 3.2.1 Potential Category Analysis of Financial Toxicity Development Trajectory in Pancreatic Cancer Patients

Based on the LCGM, we analyzed the potential development trajectories of financial toxicity in pancreatic cancer patients. We initiated the fitting process with one latent class and progressively increased the number of classes. A total of five models were fitted in this study, with the specific fitting indicators detailed in Table 2. Through comparison and analysis, we ultimately determined that the three‐latent‐class model provided the best fit. The specific reasons are as follows: Firstly, BIC and aBIC values for the three‐latent‐class model were lower compared to those for the one‐ and two‐latent‐class models, indicating that the three‐latent‐class model had an advantage in fitting the data. Secondly, the entropy value of the three‐latent‐class model was ≥ 0.80, indicating a significant degree of discrimination between the classes. Additionally, this study employed the LMR and BLRT likelihood ratio tests to further validate the fit of the three‐latent‐class model. The results showed that both test indicators were statistically significant (*p* < 0.05), further confirming the superiority and stability of the three‐latent‐class model. Finally, considering that the LMR values for the four and five‐latent‐class models were > 0.05, indicating poor model fit, we conclude that the three‐latent‐class model provides the best fit for the development trajectory of Financial toxicity in pancreatic cancer patients.

**TABLE 2:** Potential category model fitting results of financial toxicity development trajectory in 259 patients with pancreatic cancer.

Model	LL	AIC	BIC	aBIC	Entropy	LMR	BLRT	Sample proportion (%)
Class1	−2400.61	4813.23	4834.57	4815.54	/	/	/	/
Class2	−2200.92	4419.84	4451.85	4423.32	0.81	0.0075	< 0.05	60.62/39.38
Class3	−2060.06	4144.12	4186.80	4148.76	0.91	0.0001	< 0.05	19.69/56.37/23.94
Class4	−2023.13	4076.27	4129.62	4082.06	0.86	0.2549	< 0.05	42.09/14.67/25.87/17.37
Class5	−1984.67	4005.35	4069.37	4012.30	0.89	0.1351	< 0.05	25.10/13.51/40.93/3.48/16.98

#### 3.2.2 Characteristic Analysis of Three Categories of Financial Toxicity in Patients With Pancreatic Cancer

**FIGURE 1:**
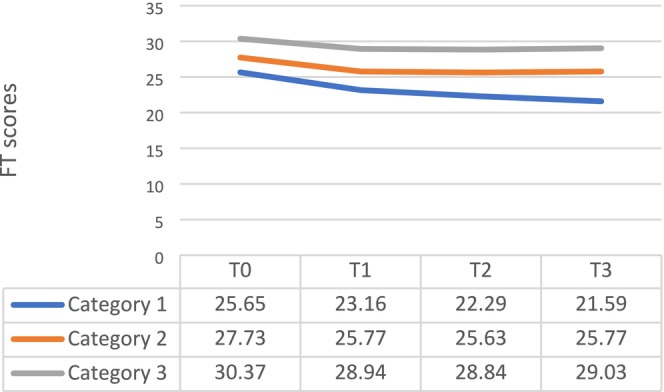
Trajectory of Financial toxicity in pancreatic cancer patients.

The model fitting results revealed three distinct development curves for financial toxicity among pancreatic cancer patients, corresponding to three categorical trajectories as shown in Figure 1. Based on the variation in patients' financial toxicity scores at different time points across these three categorical trajectories, they were named accordingly. The blue curve (Category 1) was designated as the High‐Risk‐Financial toxicity increasing group (19.69%). The orange curve (Category 2) was named the Moderate‐Risk‐Financial toxicity stable group (56.37%). Lastly, the gray curve (Category 3) represented the Low‐Risk‐Financial toxicity stable group (23.94%). Based on the results of this model fitting, it can be seen that the financial toxicity for some pancreatic cancer patients remains relatively stable in its development trend. However, there is a portion of pancreatic cancer patients who urgently require attention, as they have a lower financial toxicity score at the T0 time point and show a trend of continuous decline.

### 3.3 Analysis of Factors Influencing the Development Trajectory of Financial Toxicity in Pancreatic Cancer Patients

#### 3.3.1 Single Factor Analysis of Financial Toxicity Trajectory in Patients With Pancreatic Cancer

Based on univariate analysis, it was found that sociodemographic factors, disease‐related factors, medical expenditure factors, and self‐efficacy of pancreatic cancer patients all influenced the development of their financial toxicity. The statistically significant independent variable indicators include: age, employment status, monthly household income, quantity of children, marital status, ACCI, length of stay, loan, the main breadwinner of the family, total out‐of‐pocket medical expenses, distance to hospital, self‐efficacy (T3), as shown in Table 3.

**TABLE 3:** Results of single factor analysis of socio‐demographic data of patients with different financial toxicity development tracks.

Variable	High‐risk‐financial toxicity increasing group (*n* = 51)	Moderate‐risk‐financial toxicity stable group (*n* = 146)	Low‐risk‐financial toxicity stable group (*n* = 62)	Test value	*p*
*Gender*					
Male	33 (64.7%)	84 (57.5%)	29 (46.8%)	3.843	0.146
Female	18 (35.3%)	62 (42.5%)	33 (53.2%)		
*Age*					
< 60	32 (62.7%)	62 (42.5%)	0 (0.0%)	77.276	< 0.001
60–80	19 (37.3%)	83 (56.8%)	54 (87.1%)		
> 80	0 (0.0%)	1 (0.7%)	8 (12.9%)		
*Profession*					
Farmer	9 (17.6%)	37 (25.3%)	13 (21.0%)	5.463	0.467
Work	29 (56.9%)	79 (54.1%)	30 (48.4%)		
Cadre	0 (0.0%)	3 (2.1%)	3 (4.8%)		
Others	13 (25.5%)	27 (18.4%)	16 (21.6%)		
*Employment status*					
Full‐time job	19 (37.3%)	32 (21.9%)	3 (4.8%)	24.894	< 0.001
Part‐time job	0 (0.0%)	6 (4.1%)	2 (3.2%)		
Retried	16 (31.4%)	68 (46.6%)	41 (66.1%)		
Unemployed	16 (31.4%)	40 (27.4%)	16 (25.8%)		
*Monthly household income (yuan/month)*					
< 5000	26 (50.1%)	18 (12.3%)	0 (0.0%)	102.299	< 0.001
5000–9999	22 (43.1%)	82 (56.2%)	13 (21.0%)		
10,000–14,999	3 (5.9%)	34 (23.3%)	37 (59.7%)		
≥ 15,000	0 (0.0%)	12 (8.2%)	12 (19.4%)		
*Quantity of children*					
0	1 (2.0%)	1 (0.7%)	1 (1.6%)	10.505	0.017
1	27 (52.9%)	68 (46.6%)	17 (27.4%)		
≥ 2	23 (45.1%)	77 (52.7%)	44 (71.0%)		
*Primary caregiver*					
Wife/husband	27 (52.9%)	66 (45.2%)	19 (30.6%)	10.941	0.056
Sons and daughters	20 (39.2%)	73 (50.0%)	40 (64.5%)		
Nursing workers	0 (0.0%)	3 (2.1%)	0 (0.0%)		
Others	4 (7.8%)	4 (2.7%)	3 (4.8%)		
*Education level*					
Primary and below	13 (25.5%)	57 (39.0%)	18 (29.0%)	8.854	0.355
Junior high school	22 (43.1%)	35 (24.0%)	19 (30.6%)		
Junior high school	9 (17.6%)	31 (21.2%)	14 (22.6%)		
University	7 (13.7%)	22 (15.1%)	11 (17.7%)		
Postgraduate and above	0 (0.0%)	1 (0.7%)	0 (0.0%)		
*Marital status*					
Spinsterhood	1 (2.0%)	0 (0.0%)	0 (0.0%)	15.164	0.006
Married	48 (94.1%)	132 (90.4%)	47 (75.8%)		
Divorced	0 (0.0%)	4 (2.7%)	2 (3.2%)		
Widowed	2 (3.9%)	10 (6.8%)	13 (21.0%)		
*Housing type*					
Live with spouse	23 (45.1%)	76 (52.1%)	32 (51.6%)	5.387	0.201
Live with children	26 (50.1%)	70 (47.9%)	29 (46.8%)		
Others	2 (3.9%)	0 (0.0%)	1 (1.6%)		
*Distance to hospital (km)*					
< 100	3 (5.9%)	67 (45.9%)	40 (64.5%)	97.318	< 0.001
100–500	28 (54.9%)	77 (52.7%)	22 (35.5%)		
> 500	20 (39.2%)	2 (1.4%)	0 (0.0%)		
*Payment methods*					
New rural cooperative medical(ncm)	18 (35.3%)	55 (37.7%)	23 (37.1%)	8.507	0.353
Medical insurance for urban employees	25 (49.0%)	70 (47.9%)	35 (56.5%)		
The medical insurance for urban residents	8 (15.7%)	20 (13.7%)	3 (4.8%)		
Poverty relief insurance	0 (0.0%)	1 (0.7%)	0 (0.0%)		
Self‐paying	0 (0.0%)	0 (0.0%)	1 (1.6%)		
*Commercial insurance*					
Yes	7 (13.7%)	21 (14.4%)	14 (22.6%)	2.442	0.295
No	44 (86.3%)	125 (85.6%)	48 (77.4%)		
*Loan*					
Yes	11 (21.6%)	11 (7.5%)	0 (0.0%)	17.145	< 0.001
No	40 (78.4%)	135 (92.5%)	62 (100.0%)		
*The main breadwinner of the family*					
Yes	35 (68.6%)	79 (54.1%)	0 (0.0%)	67.319	< 0.001
No	16 (31.4%)	67 (45.9%)	62 (100.0%)		
*Cancer staging*					
1–2	29 (56.9%)	82 (56.2%)	35 (56.5%)	0.008	0.996
3–4	22 (43.1%)	64 (43.8%)	27 (43.5%)		
*Operation history*					
Yes	19 (37.7%)	56 (38.4%)	32 (51.6%)	3.586	0.166
No	32 (62.7%)	90 (61.6%)	30 (48.4%)		
*Treatment on initial admission*					
DP	16 (31.4%)	38 (26.0%)	14 (22.6%)	5.307	0.505
WP	20 (39.2%)	60 (41.1%)	24 (38.7%)		
TP	0 (0.0%)	3 (2.1%)	0 (0.0%)		
Others	15 (29.4%)	45 (30.8%)	24 (33.9%)		
*Treatment after discharge*					
0 ~ 1 (chemotherapy/radiotherapy/immunology/targeting/tcm only)	49 (96.1%)	142 (97.3%)	60 (96.8%)	0.175	0.916
≥ 2	2 (3.9%)	4 (2.7%)	2 (3.2%)		
*Enter ICU or not*					
Yes	12 (23.5%)	36 (24.7%)	14 (22.6%)	0.109	0.947
No	39 (76.5%)	110 (73.5%)	48 (24.4%)		
*Number of complications*					
0	24 (47.1%)	77 (52.7%)	37 (59.7%)	6.057	0.186
1 ~ 2	23 (45.1%)	60 (41.1%)	25 (40.3%)		
> 2	4 (7.8%)	9 (6.2%)	0 (0.0%)		
Length of stay (days)	18.76 ± 1.051	16.70 ± 0.524	15.10 ± 0.970	3.953	0.020
ACCI	3.10 ± 0.173	2.26 ± 0.108	3.27 ± 0.144	17.943	< 0.001
*Total out‐of‐pocket medical expenses (yuan)*					
< 50,000	1 (2.0%)	32 (21.9%)	20 (32.3%)	67.608	< 0.001
50,000–100,000	27 (52.9%)	107 (73.3%)	39 (62.9%)		
> 100,000	23 (45.1%)	7 (4.8%)	3 (4.8%)		
*Self‐efficacy (t3)*	21.18 ± 0.232	22.93 ± 0.127	25.37 ± 0.340	94.147	< 0.001

### 3.4 Multivariate Logistic Regression Analysis of Financial Toxicity Trajectory in Pancreatic Cancer Patients

The categorical variables among the independent variables were assigned values, and the continuous variables were directly included in the model as covariates, as detailed in Table 4.

**TABLE 4:** Variable assignment.

Variable name	
*Dependent variable*	
Financial toxicity development trajectory is subcomponent type	1 = High‐Risk‐Financial toxicity increasing group; 2 = Moderate‐Risk‐Financial toxicity stable group; 3 = Low‐Risk‐Financial toxicity stable group
*Independent variable*	
Age	1 = < 60; 2 = 60–80; 3 = > 80
Employment status	1 = full‐time job; 2 = part‐time job; 3 = Retried; 4 = Unemployed
Monthly household income (yuan/month)	1 = < 5000; 2 = 5000–9999; 3 = 10,000–14,999; 4 = ≥ 15,000
*Variable name*	
Quantity of children	1 = 0; 2 = 1; 3 = ≥ 2
Marital status	1 = spinsterhood; 2 = Married; 3 = divorced; 4 = widowed
Loan	1 = yes; 2 = no
The main breadwinner of the family	1 = yes; 2 = no
Total out‐of‐pocket medical expenses (yuan)	1 = < 50,000; 2 = 50,000–100,000; 3 = > 100,000
Distance to hospital (km)	1 = < 100; 2 = 100–500; 3 = > 500
*Concomitant variable*	
Self‐efficacy	Treated as the original value
ACCI	Treated as the original value
Length of stay	Treated as the original value

In multivariate logistic regression analysis, it was found that self‐efficacy, total out‐of‐pocket medical expenses < 50,000 (yuan), total out‐of‐pocket medical expenses 50,000–100,000 (yuan), monthly household income (yuan):5000–9999, ACCI, employment status—full‐time, distance to hospital < 100 km, distance to hospital < 100–500 km influenced the development of their financial toxicity, as shown in Table 5 (only statistically significant indicators were reported).

**TABLE 5:** Multivariate logistic regression analysis of factors influencing the development trajectory of financial toxicity in patients with pancreatic cancer.

Variable	*B*	*Wald*	OR	95% CI	*p*
*High‐risk‐financial toxicity increasing group* ^a^					
Self‐efficacy	−1.028	7.353	0.358	0.170–0.752	0.007
Total out‐of‐pocket medical expenses < 50,000 (yuan)	−7.437	7.682	0.001	0.00003–0.113	0.006
*Moderate‐Risk‐Financial toxicity stable group* ^a^					
Self‐efficacy	−1.137	15.998	0.321	0.184–0.560	< 0.001
Monthly household income (yuan): 5000–9999	5.726	5.779	306.873	2.879–32710.005	0.016
*Moderate‐risk‐financial toxicity stable group* ^b^					
ACCI	−1.144	14.110	0.319	0.175–0.597	< 0.001
Employment status‐full‐time	−1.771	3.930	0.170	0.030–0.980	0.047
Total out‐of‐pocket medical expenses 50,000‐100,000 (yuan)	1.908	4.325	6.738	1.116–40.677	0.038
Distance to hospital < 100 km	4.689	11.688	108.778	7.396–1599.792	0.001
Distance to hospital < 100–500 km	2.605	5.285	13.529	1.468–124.663	0.022

^a^ The Low‐Risk‐Financial toxicity increasing group is taken as the reference class.

^b^ The High‐Risk‐Financial toxicity increasing group is taken as the reference class.

## 4 Discussion

Our study utilized the Latent Class Growth Mixture (LCGM) model to delineate heterogeneous trajectories of financial toxicity among pancreatic cancer patients in China, revealing critical insights into temporal financial stress patterns. The high‐risk group exhibited a progressive decline in COST‐PROM scores, aligning with trends observed in other low‐ and middle‐income countries (LMICs) such as India and Vietnam, where cancer‐related financial toxicity intensifies over time due to fragmented healthcare financing systems [34, 35]. Notably, China's recent medical insurance reforms, including the expansion of critical illness insurance in 2020, have reduced out‐of‐pocket costs for some patients, yet gaps persist—particularly for high‐cost therapies like pancreatic cancer treatment. This mirrors challenges in countries like South Africa, where inequitable coverage exacerbates disparities in financial toxicity trajectories [36].

Multivariate analysis identified full‐time employment and travel distance to hospitals as key predictors of financial toxicity. In China, full‐time workers face substantial income loss during treatment due to limited sick leave policies [37]—a issue also documented in America's cancer workforce studies [38]. Comparatively, Thailand's universal coverage system mitigates employment‐related financial shocks through wage compensation schemes [39], suggesting policy refinements China could adopt. China's ongoing efforts to strengthen primary care (e.g., “Healthy China 2030”) aim to reduce cross‐regional care‐seeking, but implementation remains uneven, highlighting the need for region‐specific interventions [40].

Self‐efficacy emerged as a protective factor, consistent with global studies demonstrating its role in buffering financial stress [40]. For instance, US‐based interventions leveraging patient navigation programs improved self‐efficacy and reduced costs [41], offering a model for China. Additionally, our use of the ACCI score to stratify comorbidity risks underscores the value of standardized comorbidity assessment in LMICs [42]. Lower ACCI scores correlated with moderate‐risk trajectories, suggesting that healthier patients may better navigate financial challenges.

## 5 Strength and Limitation

Our research design—tracking patients at four time points (upon admission, at discharge, 3 months post‐discharge, and 6 months post‐discharge)—captured critical phases of financial toxicity evolution, reflecting China's treatment reimbursement cycles and regional reimbursement timelines. District‐level trajectory analysis revealed urban–rural divergences, consistent with China's uneven healthcare resource distribution. Future studies should compare outcomes across provinces with varying insurance policies (e.g., eastern vs. western China) and evaluate the impact of recent policies like DRG pilots on financial toxicity.

## 6 Conclusion

In conclusion, financial toxicity in cancer patients is a complex and important issue that requires in‐depth study from multiple perspectives. Through continuous improvement of research methods and expansion of research scope, we can better understand the nature and influencing factors of this problem and provide better financial support and medical security for cancer patients.

## Author Contributions

**Li Xiaoxuan:** data curation (equal), investigation (equal), project administration (equal), writing – original draft (equal). **Zhang Shuo:** investigation (equal). **Li Shanshan:** writing – review and editing (equal). **Yu Mengxia:** investigation (equal). **Yao Tianying:** writing – review and editing (equal). **Shen Yuxin:** data curation (equal). **Li Jiang:** methodology (equal), validation (equal). **Chen Mingxia:** software (equal), writing – original draft (equal), writing – review and editing (equal).

## Conflicts of Interest

The authors declare no conflicts of interest.

## Data Availability

The data that support the findings of this study are available from the corresponding author upon reasonable request.
